# Carbon quantum dots shuttle electrons to the anode of a microbial fuel cell

**DOI:** 10.1007/s13205-016-0552-1

**Published:** 2016-10-25

**Authors:** A. S. Vishwanathan, Kartik S. Aiyer, L. A. A. Chunduri, K. Venkataramaniah, S. Siva Sankara Sai, Govind Rao

**Affiliations:** 1 Department of Biosciences, Sri Sathya Sai Institute of Higher Learning, Prasanthi Nilayam, Puttaparthi, 515134 Andhra Pradesh India; 2 Department of Physics, Sri Sathya Sai Institute of Higher Learning, Prasanthi Nilayam, Puttaparthi, 515134 Andhra Pradesh India; 3 Center for Advanced Sensor Technology and Department of Chemical, Biochemical and Environmental Engineering, University of Maryland Baltimore County, Baltimore, MD 21250 USA

**Keywords:** Carbon quantum dots, DREAM assay, Electron mediator, Microbial fuel cell, Methylene blue

## Abstract

Electrodes based on graphite, graphene, and carbon nanomaterials have been used in the anode chamber of microbial fuel cells (MFCs). Carbon quantum dots (C-dots) are a class of versatile nanomaterials hitherto not reported in MFCs. C-dots previously synthesized from coconut husk were reported to possess hydroxyl and carboxyl functional groups on their surface. The presence of these functional groups on a carbon matrix conferred on the C-dots the ability to conduct and transfer electrons. Formation of silver nanoparticles from silver nitrate upon addition of C-dots confirmed their reducing ability. DREAM assay using a mixed microbial culture containing C-dots showed a 172% increase in electron transfer activity and thus confirmed the involvement of C-dots in supplementing redox activity of a microbial culture. Addition of C-dots as a suspension in the anode chamber of an MFC resulted in a 22.5% enhancement in maximum power density. C-dots showed better performance as electron shuttles than methylene blue, a conventional electron shuttle used in MFCs.

## Introduction

Microbial fuel cells (MFCs) are microbe-catalyzed electrochemical systems that can breakdown organic compounds in wastewater and harness the electrons produced in the process. The anode chamber of an MFC containing microbes and the substrate (typically in the form of wastewater) is maintained under anaerobic conditions to facilitate the uptake of electrons by the electrode. The protons produced in the process move across a selectively permeable membrane to the cathode chamber containing the terminal electron acceptor. MFCs present a unified solution to the global environmental issues of wastewater management and clean energy production.

Transfer of electrons from microbes to the anode is a critical step in performance of an MFC (Rabaey et al. 2004). Inefficient electron transfer has been an impediment to scaling up MFCs for practical real-world applications (Yuan et al. 2011). Electron transfer can take place directly between microbes and the electrode or via naturally produced or artificially added electron mediators (Schröder 2007). It is essential to understand the mechanism of electron transfer to and from the small, insoluble molecules that function as electron shuttles (Stams et al. 2006) and mediate electron transfer in MFCs.

Characteristics of the anode, which determine the extent of harvest and utilization of electrons, have been described by Xie et al. (2012). The anode provides a surface for colonization of microbes and serves as a conductor of electrons released from the oxidized substrates. Carbon is the most preferred anode material due to its versatility, conductivity and biocompatibility (Logan 2008). Different forms of carbon nanomaterials have been used as anode in MFCs (Ghasemi et al. 2013) to improve performance by providing a larger surface to volume ratio.

Carbon quantum dots (C-dots) are a class of “benign, abundant, and inexpensive” (Li et al. 2012) carbon nanomaterials, which have been used as sensors and catalysts in the areas of biomedicine and optronics (Wang and Hu 2014) but are hitherto unexplored in MFCs. The inherent ability of C-dots to donate and accept electrons forms the basis of the present study. This communication demonstrates the role of previously described (Chunduri et al. 2016) C-dots in microbial electron transfer and their application as an electron shuttle in the anode chamber of an MFC.

## Materials and methods

### Synthesis and characterization of C-dots

Coconut husk procured from the local market was washed twice and dried before using for synthesis of C-dots used in this study. C-dots were synthesized in a single-step hydrothermal carbonization from coconut husk by following the procedure reported by Chunduri et al. (2016).

10-gm coconut husk was added to 120 ml of double distilled water in a 200-mL Teflon lined autoclave and heated at 200 °C for 3 h. The reaction vessel was allowed to cool to room temperature and the C-dots were collected by removing the larger particles through centrifugation. The supernatant was passed through a 0.2-µm filter to eliminate micron sized particles, and the filtrate was lyophilized. A dispersion of the solid C-dots in water at a concentration of 0.5 mg ml^−1^ was used as the stock solution for further experiments. The C-dots were characterized for their size on JEOL, 2100F transmission electron microscope (TEM) operated at 200 kV. Samples for TEM analysis were prepared by drop casting the C-dots dispersion onto carbon-coated copper grids and drying them under vacuum overnight.

### Microbial culture

A mixed culture of microbes was initiated from sludge of a sewage treatment plant located in the nearby Prasanthi Nilayam Township and maintained in a nutrient medium as described in Beecroft et al. (2012). Microbial communities in sewage treatment systems predominantly comprise members of Beta-, Alpha-, and Gamma-proteobacteria, Bacteroidetes, and Actinobacteria, among many others (Wagner and Loy 2002).

### Reducing ability of C-dots and DREAM assay

A dispersion of C-dots in distilled water (0.5 mg ml^−1^) was added to 5.88-mM silver nitrate solution, and wavelength scan was performed from 250 to 600 nm using a spectrophotometer (Shimadzu) to determine reducing ability of C-dots based on formation of silver nanoparticles. Dye reduction-based electron transfer activity monitoring (DREAM) assay (Vishwanathan et al. 2015) was carried out for the mixed microbial culture with C-dots (0.02 mg ml^−1^) and without C-dots to assess electron transfer activity. A simple growth curve experiment confirmed that the microbial culture was in log phase at the time of performing the assay. A dispersion of C-dots in sterile, distilled water (without microbes) was used as control sample to ascertain whether the C-dots could decolourize methylene blue by themselves.

### MFC construction and characterization

A customized, multi-chambered MFC (Bright Glassworks, Bengaluru, India) having four anode chambers and a common cathode chamber was used to study the role of C-dots as electron shuttles. The 1000-ml central cathode chamber had a 24 cm^2^ carbon cloth electrode and air-sparged tap water as the catholyte. Each anode chamber, having a working volume of 200 ml and a carbon cloth electrode with surface area of 0.5 cm^2^, was continuously stirred using a magnetic stirrer. Cation exchange membrane (Fumatech, Germany) having an area of 7 cm^2^ separated the cathode chamber from each of the anode chambers. The MFC was connected to a data acquisition system (LabJack U12) for voltage and current measurements. Polarization and power density curves were generated using 330 to 8.2 kΩ resistors sequentially as external load for characterizing performance of the MFC. Reported power and current densities were normalized to surface area of the anode. The MFC experiments were carried out in batch-mode.

### C-dots as electron shuttles in the MFC

**Fig. 1:**
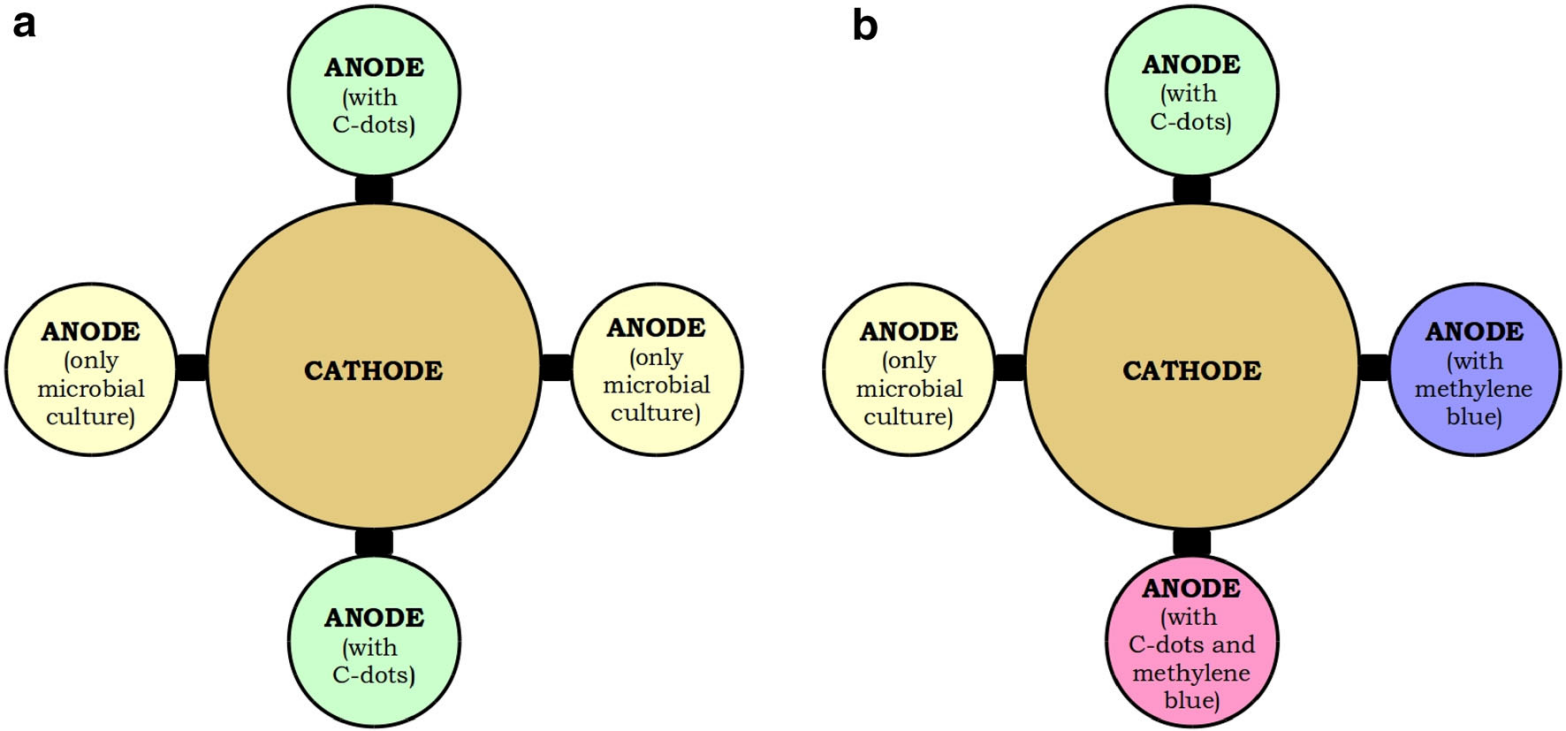
Layout of the multi-chambered MFC for the experiments **(a)** with and without C-dots and **(b)** with C-dots and methylene blue

C-dots (0.02 mg ml^−1^) were added to two anode chambers of the multi-chambered MFC containing mixed microbial culture. The other two chambers contained the same mixed microbial culture, without C-dots. In another set of experiments, three of the anode chambers containing mixed microbial culture were supplemented with C-dots (0.02 mg ml^−1^), methylene blue (50 mM), and a combination of C-dots and methylene blue. The fourth chamber contained only the microbial culture without any additional electron shuttle. Figure 1 shows a graphic representation of the experimental layout.

## Results

### Structural and functional characterization of C-dots

**Fig. 2:**
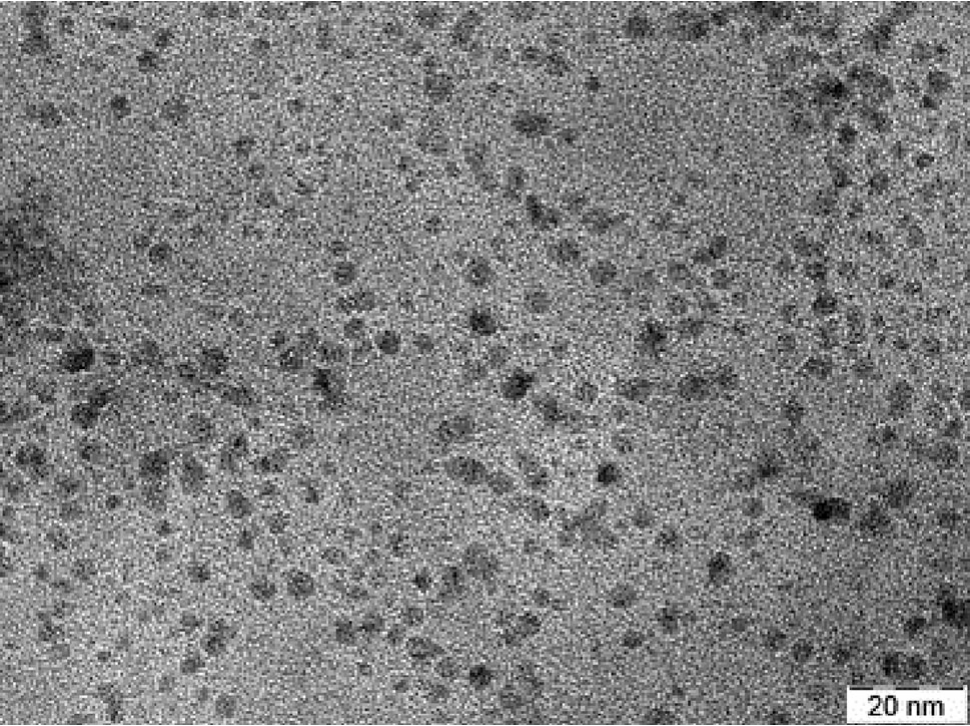
TEM image of the C-dots used in this study

The C-dots synthesized from coconut husk by hydrothermal treatment were monodisperse in nature and no aggregation was observed. TEM image (Fig. 2) showed that the C-dots were spherical in shape and lesser than 10 nm in diameter. Detailed structural and functional characterization of the C-dots used in this study was performed and described previously by Chunduri et al. (2016). X-ray diffraction spectrum confirmed the crystalline structure and Raman spectrum indicated the graphitic constitution of the C-dots. Fourier transform infra red (FTIR) spectrum of the C-dots revealed the presence of carboxyl and hydroxyl groups.

### Reducing ability and electron transfer activity of C-dots

Reduction of silver nitrate solution to silver nanoparticles by the C-dots was attested by an absorption peak at 430 nm accompanied by appearance of a typical brownish yellow color. This characteristic feature of silver nanoparticles in solution concurred with results presented by Song and Kim (2009). Spectrometric validation was sufficient to experimentally confirm the reducing ability of C-dots. Detailed characterization of the silver nanoparticles was not carried out as it was beyond the scope of this study.

**Fig. 3:**
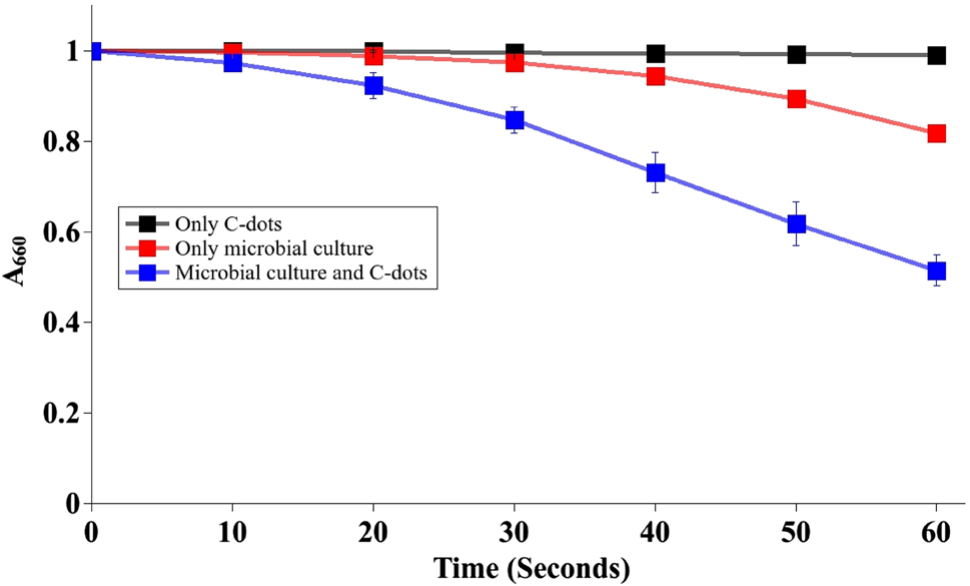
Microbial culture containing C-dots resulted in a faster rate and greater extent of decolorization of methylene blue in the DREAM assay confirming the role of C-dots as an electron mediator

Contribution of C-dots to electron transfer was assessed using the DREAM assay. Decolorization of methylene blue dye to its reduced leuco-form in the assay was directly proportional to the rate at which electrons were transferred to it by the microbes. As shown in Fig. 3, log phase microbial culture showed a 172% enhancement in dye decolorization when supplemented with C-dots. There was also a significant improvement in the kinetics of the process as seen in the markedly steeper slope after each 10-s interval during the minute-long assay. The control sample containing only C-dots in sterile, distilled water (without microbes) did not decolorize methylene blue.

### Performance of C-dots in the MFC

**Fig. 4:**
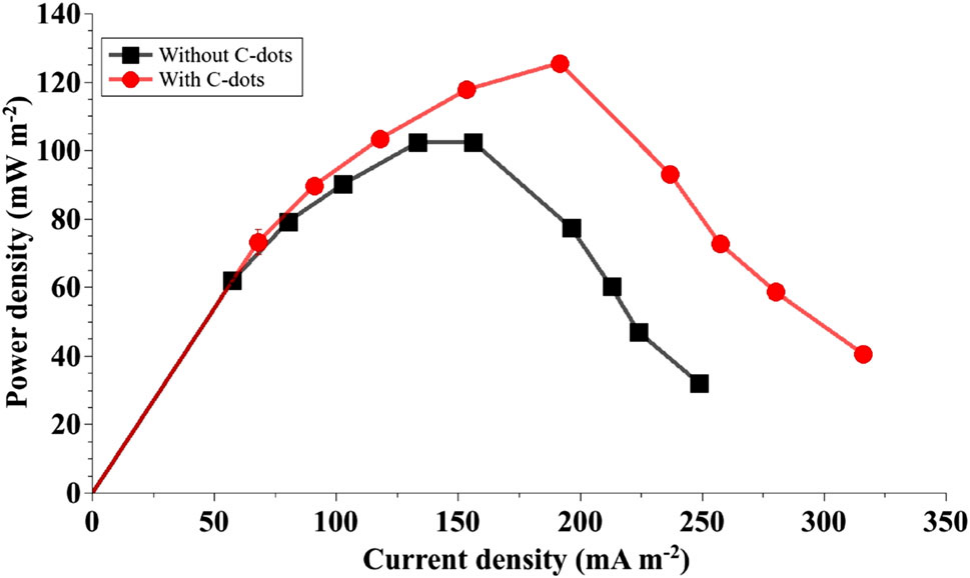
Maximum power density of a microbial fuel cell was significantly enhanced in the anode chambers supplemented with C-dots

The multi-chambered MFC facilitated the study and characterization of mixed microbial cultures with and without C-dots, in duplicate. Figure 4 shows that addition of C-dots, with their ability to supplement electron transfer activity of microbes, to the anolyte enhanced maximum power density to 126 mW m^−2^ and corresponding current density to 191 mA m^−2^ marking an increase of 22%.

**Fig. 5:**
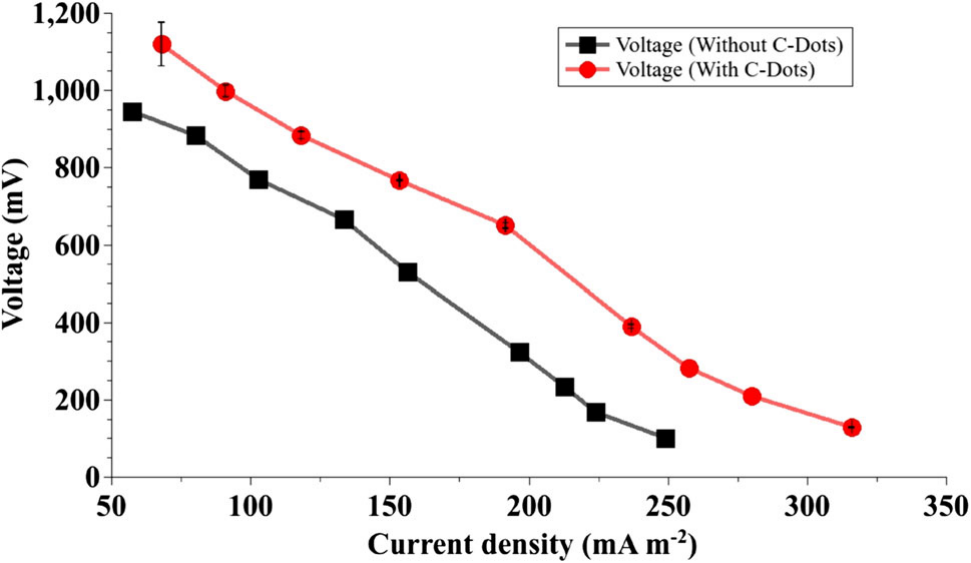
For the given values of external resistance, voltage and current densities were significantly higher in the anode chambers containing C-dots implying a decrease in overpotentials due to improved electron transfer

The anode chambers with C-dots produced higher current density compared to the ones without. Polarization curves in Fig. 5 point to the lowering of internal electrochemical losses due to improved efficiency of electron transfer.

**Fig. 6:**
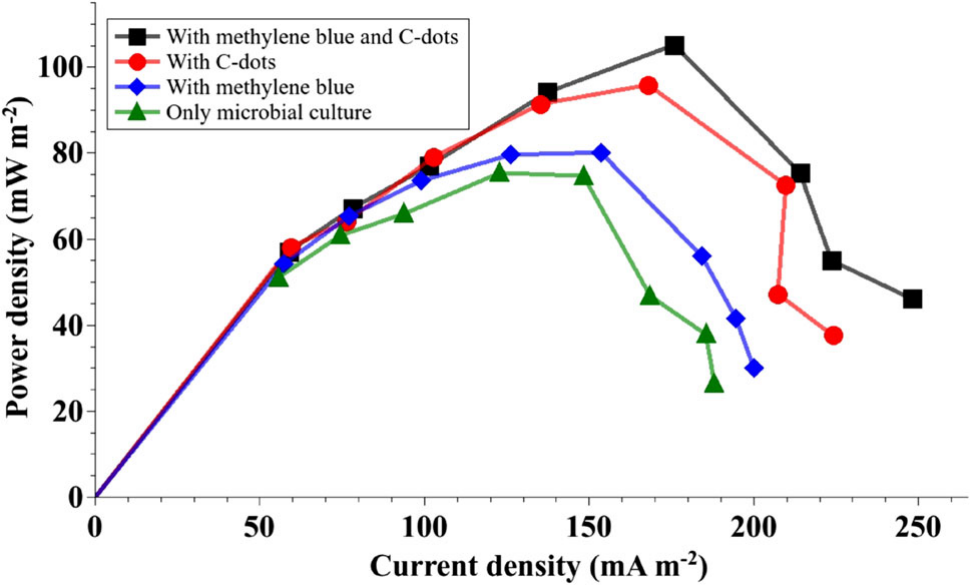
Performance of C-dots as electron shuttles was superior compared to that of methylene blue. A combination of C-dots and methylene blue resulted in higher power densities than either of them used individually

Figure 6 shows that addition of C-dots to the anolyte enhanced power density of the microbial culture by 28% as compared to the 6% increase obtained with the addition of methylene blue. A combination of methylene blue and C-dots increased the maximum power density of the MFC by 40%.

## Discussion

### C-dots possess functional groups that facilitate electron transfer

The nano-range dimensions of the C-dots provided an extensive surface area for electron transfer in the anode chamber. C-dots have been used previously as reducing agents for synthesis of gold nanoparticles and nanocomposites (Jiang et al. 2014). Due to the nature of their bonding to the carbon scaffold and the presence of a strong electronegative center, the functional groups present on their surface conferred reducing potential for the formation of silver nanoparticles from silver nitrate. C-dots rich in hydroxyl groups possess reducing property (Lu et al. 2015), while carboxyl groups are known to facilitate electron transfer by forming strong hydrogen bonds with bacterial cytochromes (Tang et al. 2011).

### C-dots supplement microbial electron transfer

DREAM assay was used to evaluate the electron transfer activity of microbes. Although the C-dots had inherent reducing potential, they were unable to decolorize methylene blue dye only by themselves. The assay conclusively demonstrated that the mixed culture of microbes used in this study possessed electron transfer activity which was further enhanced by addition of C-dots. This enhancement resulted from the participation of C-dots as electron shuttles between the microbes and the dye. We have demonstrated previously that the presence of a competitive electron acceptor with a high redox potential, such as oxygen, can hamper electron transfer to the dye during the DREAM assay (Vishwanathan et al. 2015). The small size of the conducting C-dots and the presence of electron-withdrawing and electron-donating functional groups on their surface qualified their use as electron shuttles. The role of these functional groups in surface passivation also consequently supported their use as a stable dispersion in the anode chamber of an MFC.

### C-dots shuttle electrons in the anode chamber of an MFC

Electron transfer from microbes to the anode is the keystone of MFCs. Small-sized molecules that get readily reduced and oxidized have been used as mediators or electron shuttles in MFCs. Research on electron mediators in MFCs has not evoked extensive interest since the report on direct transfer of electrons by microbes to the anode of an MFC (Kim et al. 2002). Only microbes that do not require exogenous electron shuttles or mediators have harbored interest in MFC research (Logan 2012). Moreover, artificial mediators have not been preferred on account of their instability and toxicity (Du et al. 2007). The demonstration of C-dots as electron shuttles in this study is for improving performance efficiency and not as a prerequisite for electron transfer. Moreover, the C-dots used in this study are biocompatible and do not have detrimental effect on microbial growth.

Performance of an MFC is commonly assessed in terms of electrochemical overpotentials as depicted by power density and polarization curves. Activation overpotentials arise due to energy loss in catalyzing redox reactions and during electron transfer from microbes to the electrode; ohmic overpotentials are a result of resistance to movement of electrons and mass transfer overpotentials are attributed to concentration gradients formed due to ineffective circulation of reactants in the reaction vessel (Logan 2008). Addition of the C-dots dispersion in the anode chamber and continuous stirring of the anolyte enhanced electron transfer by improving conductivity of the anolyte and facilitating collection of electrons from different regions of the anode chamber. Activation and ohmic overpotentials were minimized by the increased surface area provided by the C-dots. Sharma et al. (2008) reported similar findings in their study on the effect of nanofluids in an MFC. Logan et al. (2006) described a shift in the power density and polarization curves upon reduction of ohmic overpotentials. The harvest of electrons from a mixed microbial culture in an MFC increased in the presence of C-dots.

Methylene blue, an inexpensive redox dye that is soluble in the anolyte, is less toxic compared to other such redox mediators (Popov et al. 2012) and is known to enhance power and current density in MFCs (Daniel et al. 2009). Adsorption of methylene blue by carbon nanotubes has been studied previously (Yan et al. 2005; Shahryari et al. 2010). Detailed electrochemical studies could reveal more clues about the synergistic effect of the combination of C-dots and methylene blue in electron transfer observed in this study.

Microbial electrochemical systems, in general, have diverse applications, such as wastewater treatment (Lefebvre et al. 2011), biohydrogen production (Cheng and Logan 2007), desalination (Cao et al. 2009), and metal recovery from leachates (Modin et al. 2012) among many others. Due to extensive electrochemical losses, current densities produced by these systems are very low for applications in terms of power production (Clauwaert et al. 2008). However, a practical application of an MFC as power supply source was described by Tender et al. (2008) using a specialized voltage conditioner and capacitor. High-performance electrodes are also associated with increased costs.

Such bioelectrochemical systems can be scaled up for practical applications by minimizing electrochemical losses, enhancing performance efficiency and lowering operation costs. This study using C-dots synthesized from locally available coconut husk as electron shuttles is a step forward in this direction. The results of this study have the potential to unravel new facets of microbial electron transfer driven by interactions between carbon nanostructures and microbes.
